# Transdermal Scopolamine for Treatment-Resistant Major Depressive Disorder

**DOI:** 10.7759/cureus.68447

**Published:** 2024-09-02

**Authors:** Mitchell B Liester, Delia S Shash

**Affiliations:** 1 Psychiatry, University of Colorado School of Medicine, Colorado Springs, USA; 2 Obstetrics and Gynecology, University of Arizona, Phoenix, USA

**Keywords:** glutamate synaptic transmission, hyoscine, brain-derived neurotrophic factor, rapid-acting antidepressant drug, treatment-resistant depression

## Abstract

Conventional antidepressants are useful in the treatment of major depressive disorder (MDD) but are limited by their delayed onset of action and lack of adequate therapeutic response in approximately one-third of patients. This has led to a quest for faster-acting and more effective antidepressants. Scopolamine exhibits rapid antidepressant effects when administered intravenously. We report a case of a female with treatment-resistant depression (TRD) who responded to transdermal scopolamine. She had a rapid (within three days) and sustained (119 days) response to transdermal scopolamine. Further research into the use of transdermal scopolamine for the treatment of depression is recommended.

## Introduction

Major depressive disorder (MDD) is a common psychiatric disorder with a lifetime prevalence of 16% in the United States [1]. The currently available antidepressant medications are ineffective in about one-third of patients [2] and their onset of action is often delayed for weeks or months [3]. A subset of patients fail to respond to an adequate dose and duration of treatment with at least two antidepressant medications. These patients are described as having “treatment-resistant depression” (TRD) [4]. Medicines that act rapidly are referred to as “rapid-acting antidepressant drugs” (RAADs). Scopolamine is a RAAD that has been suggested as a potential therapy for TRD [5]. Scopolamine (aka hyoscine) is a tropane alkaloid derived from plants in the nightshade family (Solanaceae) that acts as a non-selective antagonist of muscarinic acetylcholine receptors (mAChRs) [6]. It has exhibited rapid antidepressant effects in both preclinical and clinical studies [7-8]. However, scopolamine is not yet approved by the FDA for the treatment of depression.

Scopolamine’s antidepressant effects are believed to result from the blockade of mACHRs on gamma-aminobutyric acid (GABA) interneurons, which triggers the rapid (i.e., within 10 minutes) release of glutamate from presynaptic glutamatergic neurons in the medial prefrontal cortex (mPFC). Glutamate then binds to α-amino-3-hydroxy-5-methyl-4-isoxazolepropionic acid (AMPA) receptors, which stimulates voltage-dependent calcium channels (VDCCs), resulting in an influx of calcium into postsynaptic glutamatergic neurons. This influx of calcium increases the release of brain-derived neurotrophic factor (BDNF), a protein member of the neurotrophin family of growth factors. BDNF binds to tropomyosin receptor kinase B (TrK-B) receptors, resulting in increased activation of the mammalian target of the rapamycin complex 1 (mTORC1) signaling pathway. Activation of this pathway stimulates neurogenesis and synaptogenesis, resulting in improvement in depression [9]. 

When administered at a dose of 4.0 µg/kg intravenously, scopolamine produces rapid and robust antidepressant effects [8]. These effects are often seen within three days of treatment and persist for more than two weeks following the discontinuation of treatment. Scopolamine is generally well tolerated and its adverse effects are typically mild and transient. A search of both PubMed and Google Scholar found no reports describing the use of transdermal scopolamine for the treatment of depression, making this report a rare and unique one.

## Case presentation

The patient was a 68-year-old female with a history of TRD, who had previously tried and failed trials of adequate duration and dosage with multiple antidepressant medications including amitriptyline, bupropion ER, imipramine, fluvoxamine, levomilnacipran (Fetzima), vilazodone (Viibryd), and vortioxetine (Trintellix). She also did not respond to amphetamine-dextroamphetamine (Adderall), modafinil (Provigil), armodafinil (Nuvigil), or l-methylfolate. After she was diagnosed with Parkinson’s Disease (PD), the patient was prescribed carbidopa/levodopa, which ameliorated her tremors but did not affect her depression. Selegiline was added, but after one month, her depressive symptoms had not improved. The Beck Depression Inventory (BDI) revealed a score of 32, placing the patient in the severe range of depression. A trial of transdermal scopolamine was discussed with the patient, and she consented. The treatment consisted of a scopolamine 1 mg transdermal patch applied behind the ear, and the patch was replaced with a new patch every fourth day. 

Three days after initiating treatment with transdermal scopolamine, the patient noted improvement in her mood. By the sixth day of treatment, her mood was markedly better, and she experienced increased energy and improved sleep. Also, the sense of apathy and dread she experienced before scopolamine was reported to have improved by 95%. She described increased social interactions and improved concentration. She was exercising more frequently, walking for up to 60 minutes on a treadmill, and she was enjoying the exercise routine. She denied anxiety and described moderate dry mouth as the only adverse effect of the scopolamine treatment. She stated: “I feel like a normal person.” Six days after starting transdermal scopolamine, her BDI score was 5, indicating no persistent depression. 

The patient was seen for a follow-up 18 days after initiating treatment with transdermal scopolamine. She had just returned from a trip to the Caribbean where she had snorkeled, swum with rays, and sailed. She stated that she had enjoyed the trip. She completed another BDI at that time and her score was 3. Her BDI score subsequently remained at 3 or less for the next 14 weeks. Her last evaluation was on treatment day 119 and her BDI was 1 at that time.

## Discussion

This report describes a rapid antidepressant response following treatment with transdermal scopolamine in a female with severe TRD. Several confounding factors need to be considered when evaluating this patient’s clinical response to transdermal scopolamine. Firstly, selegiline was started one month before the initiation of treatment with transdermal scopolamine. Selegiline is a selective, irreversible monoamine oxidase B inhibitor (MAOI-B) with known antidepressant properties. It is possible this patient’s improvement resulted from the use of selegiline and not transdermal scopolamine. However, she had experienced no improvement in depressive symptoms for four weeks after initiating treatment with selegiline, making it unlikely that she would rapidly shift from severe depression to remission following six additional days of treatment. It is also possible that selegiline and scopolamine acted synergistically to produce a rapid antidepressant response. Further studies are needed to determine whether such a synergistic response can occur with the concurrent use of these medicines. 

Another factor to consider is whether this patient’s comorbid PD played a role in her response to transdermal scopolamine. The pathophysiology of PD includes neurodegeneration and depression is viewed by some to have a similar pathophysiologic mechanism [10]. Are individuals with PD more likely to respond to a RAAD that triggers neurogenesis and synaptogenesis than individuals who suffer from subtypes of MDD associated with different pathophysiological mechanisms, such as inflammation or monoamine depletion? Research studies examining this question are warranted. 

Another possible explanation for this patient’s improvement is a placebo response. Individuals with PD release increased levels of dopamine following the administration of a placebo [11], and the placebo response is often a confounding factor in clinical studies of antidepressant medicines [12]. Double-blind placebo-controlled studies could help clarify whether the placebo response plays a significant role in the positive antidepressant response to transdermal scopolamine. 

The use of transdermal scopolamine as a treatment for depression is associated with risks. Hence, caution must be exercised when prescribing scopolamine to a patient who is also taking other medicines with anticholinergic properties, as this increases the risk of anticholinergic side effects (e.g., xerostomia, blurred vision, and constipation). Also, the absorption of oral medications may be delayed by scopolamine treatment due to delayed gastric motility and delayed gastric emptying. Increased intraocular pressure may result from the coadministration of corticosteroids with antimuscarinics. Additionally, the concurrent administration of scopolamine and levodopa may reduce the absorption of levodopa in the small intestine due to the increased metabolism of levodopa in the stomach. 

If scopolamine is discontinued without a concomitant reduction in levodopa dosage, toxicity may occur due to increased absorption of levodopa [13]. Also, psychosis has been reported following scopolamine therapy [14] and withdrawal symptoms including dizziness, nausea, vomiting, headache, and disturbance of equilibrium can occur following discontinuation of scopolamine therapy lasting more than three days [15-16]. On the other hand, scopolamine has been safely administered continuously for three years for the prevention of seasickness without significant adverse effects [17].

## Conclusions

At least one-third of patients with MDD fail to respond to conventional antidepressants, and, when patients do respond, they often must wait for weeks to experience the antidepressant effects of these medicines. These limitations related to conventional antidepressants highlight the importance of finding novel treatments that work rapidly and are effective for TRD. We discussed a case of a patient with TRD who experienced a rapid and sustained positive response to treatment with transdermal scopolamine. Our findings, in combination with the positive results obtained from previous studies involving intravenous scopolamine, suggest that further studies are warranted to explore transdermal scopolamine’s efficacy and safety as a rapidly-acting antidepressant medication.
